# Nutritional and sensory enhancement of traditional wheat-based yeasted steamed dumplings through oat flour fortification

**DOI:** 10.3389/fnut.2025.1676290

**Published:** 2025-11-14

**Authors:** Michaela Havrlentová, Lucie Jurkaninová, Ivan Švec, Daniel Jánoška, Jana Klimáčková, Jana Hendrichová, Peter Hozlár, Soňa Gavurníková

**Affiliations:** 1 Department of Biotechnology, Faculty of Natural Sciences, University of St. Cyril and Methodius, Trnava, Slovakia; 2 National Agricultural and Food Centre – Research Institute of Plant Production, Piešťany, Slovakia; 3 Department of Food Science, Faculty of Agrobiology, Food and Natural Resources, Czech University of Life Sciences, Prague, Czechia; 4 Faculty of Food and Biochemical Technology, University of Chemistry and Technology Prague, Prague, Czechia; 5 National Agricultural and Food Centre – Research Institute of Plant Production – Research and Breeding Station, Vígľaš, Slovakia

**Keywords:** oat wholegrain flour, functional foods, *β*-d-glucans, chemical composition, sensory evaluation, correlation, principal component analysis

## Abstract

This study investigated the chemical composition and sensory acceptability of yeasted steamed wheat dumplings – a typical Central European side dish – that were fortified with wholegrain oat flours derived from two distinct cultivars – hull-less Inovec and hulled Prokop. The flours were used at 7 substitution levels, ranging from 2.5 to 25.0%. Chemical analysis of both flour mixtures and steamed dumplings revealed a significant modulation of macronutrient profiles with increasing oat inclusion. As presumed, the *β*-d-glucans content increased proportionally with the addition of oats, reaching up to 1.23% in flour mixtures (Prokop) and 0.80% in steamed dumplings (Inovec). Furthermore, enrichment by oat resulted in higher protein (up to 15.05%), lipid (up to 12.13%), and dry matter contents, while reducing starch levels. Two-factorial ANOVA attributed the most variance in nutritional traits (e.g., 87% for *β*-d-glucans) to the level of wheat flour substitution, with minor effects coming from the oat variety. The correlation analysis confirmed strong formulation-dependent trends, particularly for *β*-d-glucans (*r* = 0.972), lipids (*r* = 0.919), and proteins (*r* = 0.831), with the thermal stability of key nutrients demonstrated in all processing stages. Sensory evaluation revealed an inverse relationship between oat content and dumplings acceptability, primarily due to undesirable changes in color, aroma, and texture at higher inclusion levels. Multivariate PCA and two-way joining heatmap analysis highlighted that measurable nutritional enhancement was evident from the addition of just 2.5% oat flour, while the most favorable balance between an improved nutrient profile and retained sensory attributes was observed at inclusion levels of 10–15%. Oat Inovec-enriched samples exhibited superior protein and lipid content yet were more prone to sensory degradation at high inclusion rates. Overall, fortification with oat flours at levels of up to 10–15% is identified as an effective strategy for developing nutritionally enriched, fiber-rich yeasted steamed products with minimal sensory compromise. The findings emphasize the importance of balancing nutritional gains with sensory acceptability in the design of functional cereal-based foods.

## 1 Introduction

In recent decades, the global rise in chronic non-communicable diseases, including obesity, type 2 diabetes, cardiovascular disease, and metabolic syndrome, has been closely linked to poor diet habits and the low nutritional value of commonly consumed foods (1, 2). In response to this challenge, the concept of functional foods has gained prominence in the fields of nutrition and food technology. These foods not only offer basic nutritional value, but also specific health benefits, such as immune support, cholesterol regulation, and improvement of the gut microbiota (3, 4).

Among the bioactive components of functional foods studied most extensively are *β*-d-glucans, indigestible polysaccharides found in the cell walls of certain cereals (mainly oats and barley), fungi, yeast, and algae (5). Cereal *β*-d-glucans are linear glucose polymers linked by β-(1 → 3) and β-(1 → 4) glycosidic bonds, which endow them with distinctive physicochemical properties, including solubility, viscosity, and gel-formation ability (6–10). Viscosity is widely considered the predominant mechanism underlying its physiological effects, including delayed digestion and reduction in postprandial glycemia and cholesterol levels (10–12). Numerous studies have also demonstrated the prebiotic and anti-inflammatory properties of cereal *β*-d-glucans (13, 14). The European Food Safety Authority (EFSA) and the US Food and Drug Administration (FDA) recognize oat- and barley-derived *β*-d-glucans as beneficial compounds when consumed at a daily dose of at least 3 g (15).

The presence of β-d-glucans has been observed in the endosperm, aleurone layer, and bran of various cereals, with a notable prevalence in barley (*Hordeum vulgare* L.) and oats (*Avena sativa* L.) grains. The content varies significantly between different species and cultivars and is influenced by genetic and environmental factors (9). For example, barley and oats typically contain 3–7% (16), with some barley varieties reaching up to 11% (17). On the contrary, wheat (*Triticum aestivum* L.) and rye (*Secale cereale* L.) have less than 1% (16). Several factors, including concentration, molecular weight, and the DP3: DP4 ratio, influence the functional properties of the polysaccharide. These factors have been shown to affect viscosity and gelling capacity (6, 9, 10), as well as functionality in the plant and the food product.

Oats are widely recognized for their nutritional value, attributed to their high levels of dietary fiber and proteins, making them an important component of both human diets and livestock feed. Compared to other cereal grains, oats have relatively higher concentrations of proteins and lipids. In addition to *β*-d-glucans, the mature grain contains approximately 60% starch, 14% proteins, 7% lipids (18, 19), and metabolites such as phenolic acids, flavonoids, carotenoids, vitamin E, phytosterols, and avenanthramides with significant antioxidant activity (19, 20). Although oats contain a substantial amount of starch with the potential to elevate blood glucose levels, various bioactive constituents – such as oat *β*-d-glucans and phenolic compounds – can attenuate glycemic index responses through multiple physiological mechanisms (10, 11, 18). Furthermore, the intake of oat-derived polar lipids and proteins intake can improve hypercholesterolemia by increasing the excretion of fecal bile acids (21) and postprandial glucose and insulin responses and affect metabolic outcomes of the second meal (22). It is also widely accepted that dietary proteins can balance blood glucose by slowing the gastric emptying rate, promoting the secretion of insulin, and affecting starch digestibility (23).

The inclusion of pure oats – cultivated under controlled conditions to prevent cross-contamination with gluten-containing cereals such as wheat, rye, and barley – in a gluten-free diet offers significant nutritional advantages for individuals with celiac disease (18, 24). However, emerging evidence indicates that certain oat cultivars may express polypeptides analogous to wheat gliadins, which could generate immunogenic responses in susceptible individuals (24).

Although most oat production is used as livestock feed, oats are suitable for human consumption and have many applications, including flakes, oatmeal, and oat flour. A popular form of oat grain in the human diet is also oat bran and the whole grain of naked oats. Oats are generally consumed as whole grains or in the form of flakes, which retain a high proportion of their nutrients. Their consumption is recommended for the prevention of various diseases, including those related to digestion, metabolism, inflammation, and carcinogenesis.

The objective of this study was to design and experimentally validate the incorporation of oat flour, rich in metabolites such as *β*-d-glucans and lipids, into a culturally relevant food model – yeasted steamed wheat dumplings – to create a novel functional food in the side dish category. The goal was to develop food with a higher nutritional value than a wheat flour product and at least comparable sensory acceptance. Specifically, two varieties of oats of Slovak origin were included in the experiment, one characterized by husks on the grain and one hull-less. The chemical composition of the flour mixes and the steamed products manufactured subsequently were analyzed, as well as the innovative foods created with the addition of wholegrain oat flour into the wheat flour, sensory evaluation was performed.

This research contributes to a more profound understanding of the stability of *β*-d-glucan under real-world food processing conditions. The study also demonstrates the feasibility of enhancing traditional foods with functional components, which has the potential to broaden the application of β-d-glucans beyond conventional cereal products. This work is pertinent to innovation in the field of dietary fiber enrichment, particularly in culturally specific and underexplored food systems.

## 2 Materials and methods

### 2.1 Plant material

Two varieties of oats (*Avena sativa* L.) of Slovak provenance were used in the experiment. The plants of the variety Prokop and Inovec (further Pro and Ino) were grown in the year 2022 (April – July) in the fields of the Research and Breeding Station at Vígľaš-Pstruša, Detva, Central Slovakia (National Agricultural and Food Centre – Research Institute of Plant Production, Piešťany).

The experimental site is situated in a potato production area at 375 m above sea level, with podzolized brown earth soil. The mean annual temperature is 8.0 °C and the mean annual precipitation is 666 mm.

In the autumn before the establishment of the experiments, plowing was carried out with the incorporation of natrium-phosphorus-kalium fertilizer at a dose of 1 q/ha. Pre-sowing preparation for the experiment took place in March 2022 with the incorporation of ammonium nitrate fertilizer at a dose of 2 q/ha with skid harrows 2x. The varieties were sown in March with a Seedmatic small-plot seeder. The plot size for 1 variety was 1 × 1.4 m in four repetitions. The row spacing was 12.5 cm. During the vegetation period, the experiment was treated with the herbicide Mustang Forte at a dose of 0.8 L/ha and 2 x insecticides (sedge and cocksfoot with the Karate Zeon preparation at a dose of 0.1 L/ha). During the vegetation period, ammonium nitrate fertilization was applied at a dose of 1 q/ha at the time of full tillering. Genotypes were harvested by hand cutting at the end of July 2022. The seeds were processed on a thresher and then a cleaner and prepared for further analysis.

The yellow-hulled variety Prokop (*Avena sativa* L.) was registered in 2011. Prokop is an early-maturing oat variety with a medium height stand (107 cm) and a medium height panicle. The weight of a thousand grains is high (33.88 g). The variety is characterized by a very high yield, suitability for the mountain growing region, high volume weight, low to medium huskiness, and good resistance to diseases. The hull-less variety Inovec (*Avena sativa* var. *nuda* L.) was registered in 2017. It is an early to medium-early variety with a very low proportion of hulled grains. The variety is characterized by the highest weight of a thousand grains on the Slovak market, with a very high yield and good health.

After harvest, mature grains of hulled oat were husked. After milling of 250 g of each variety of oat to a grain thickness of 0.5 mm using Ultra Centrifugal Mill ZM300 (Retsch, Haan, Germany), both varieties were stored in sealable containers under controlled conditions (19 °C, 85% relative humidity, and darkness). The samples were used for the analysis of chemical composition and the preparation of mixed flours.

### 2.2 Binary flour mixes

Binary flour mixes were prepared by blending semi-coarse wheat flour (Vitaflora brand, Mlyn Kolárovo, a.s., Kolárovo, Slovakia; abbreviation WF) with wholegrain oat flour from the two tested varieties. Seven addition levels of oat flour (2.5, 5.0, 7.5, 10.0, 15.0, 20.0, and 25.0% w/w) were formulated, resulting in a total of 14 oat-wheat combinations plus the wheat control. Each mixture was prepared in three independent replicates of 300 g to ensure reproducibility. Of this amount, 250 g was used for dumpling preparation and 50 g for chemical analyses (nutritional parameters and dry matter determination).

The control flour used for preparing the binary mixtures was a commercial semi-coarse wheat flour (Vitaflora brand), produced and packaged by Mlyn Kolárovo, a.s. (Kolárovo, Slovakia; abbreviation WF). The gluten content (≥24% of dry matter) and falling number (≥170 s), both complied with the minimum quality requirements specified by the national standard of the Ministry of Agriculture of the Slovak Republic for milling products from wheat grain.

The oat flour was milled to a particle size of approximately 0.5 mm and had a moisture content of 10.5% prior to mixing. Mixing was performed in a laboratory blender RM800 (JAZ s.r.o., Nové Mesto n. Váhom, Slovakia) for 5 min to achieve homogeneity. The prepared flour mixtures were stored in sealed polyethylene containers under controlled conditions (19 °C, 85% relative humidity, and darkness) until further use. Coding of flour mixtures (F) was derived from the oat flour ratio and the three-letter code of the oat variety (WF for the wheat control, and F2.5Ino, F2.5Pro, …, F25Ino, F25Pro).

### 2.3 Yeasted steamed wheat dumplings preparation

The following ingredients were used in the process of the production of homemade yeasted steamed dumplings, traditionally based on wheat flour: 250.0 g flour (or flour binary mix), 60 mL pasteurized semi-skimmed cow milk, 60 mL tap water, 3.0 g salt, 1 mid-size hen egg (ca 55 g net weight), 14.0 g sunflower oil, 10.0 g compressed baker’s yeast (VIVO, Lesaffre Magyarország, Budapest, Hungary, *Saccharomyces cerevisiae* Hansen stock culture, dry matter min. 28%, packaged in 42 g cubes), and 4.0 g granulated beet sugar. The ingredients were purchased on the Slovak retain market.

A yeast pre-ferment was prepared by mixing 50 mL of water with milk, yeast, and 4.0 g sugar, and left to ferment for 15 min at 21 ± 1 °C. Then, an egg and oil were added to the remaining milk and water. Then, all these ingredients were added to the yeast preferment. The flour with all other ingredients was mixed on HL200 20-quart planetary mixer Hobart Legacy+® (Hobart Corporation, United States) with a kneading hook for 3 min at the first speed level (107 RPM) and then 5 min at the third speed level (365 RPM), producing a dough piece of 458 g. The doughs were fermented for 40 min at 21 ± 1 °C covered with a kitchen cloth until they visibly doubled in volume. After fermentation, the doughs were shaped into oblong ovals of standardized weight (ca. 300 g each) and placed on perforated baking sheets greased with sunflower oil. The shaped doughs underwent a final proof for 15 min under the same temperature conditions. Proofed doughs were steamed in an electric convection oven (Lainox Ali Group, Italy) at 100 °C for 20 min. Immediately after steaming, the dumplings were skewered to release excess steam and brushed with sunflower oil.

Each dumpling variant was prepared in triplicate (three independent batches), and each oat variety was used on a separate day, 2 days in a row. The dumplings were analyzed from a sensory perspective immediately after being removed from the convection oven, comparably to serving in homes and restaurants. In the middle part, the dumplings were cut crosswise on a stoneware plate, and 5 slices of 1 cm thickness were cut from each part. Each slice was cut crosswise in the middle and analyzed sensory. For chemical analyses, the dumplings not used for sensory evaluation were cut on a stoneware plate into cubes measuring 1.5 cm × 1.5 cm and dried at a temperature of 21 °C for 5 days in a space where air circulation was ensured. The cubes were checked 3 times a day and shaken to prevent moisture accumulation and the development of mold and other defects. After drying, they were ground to a grain thickness of 0.5 mm using Ultra Centrifugal Mill ZM300 (Retsch, Haan, Germany) and analyzed for selected nutritional parameters. The coding of the dumpling (D) variants was derived from the oat flour ratio and the three-letter code of the oat variety tested (WD for the plain wheat control, and D2.5Ino, D2.5Pro, …, D25Ino, D25Pro).

### 2.4 Sensory evaluation

In comparison with the control sample, all anomalies in dough preparation and subsequent leavening of the analyzed food products from mixed flours were monitored. Differences in the shape and structure of the dough compared to the control sample were analyzed objectively. After the dumplings were subjected to heat treatment, the parameters that included individual length, width, and weight were evaluated by 10 evaluators. The color and surface structure of the product were also analyzed visually in comparison to the control sample. The whole dumplings and those cut in half were visually evaluated, where the color of the crumb, consistency, and porosity were compared to the control sample. The taste, unusual flavors, and consistency were also analyzed by a panel of 10 evaluators (5 women and 5 men aged 15 and trained). Sensory evaluation took place in the dining room without artificial lighting. There was plenty of sunlight and similar meteorological conditions on both days. All samples prepared on the same day were evaluated at the same time and without time gaps. The samples were on white porcelain plates.

The sensory attributes evaluated within the questionnaire were as follows:

1. dumpling shape,
2. dumpling surface color,
3. dumpling smell,
4. dumpling taste,
5. dumpling porosity,
6. color of dumpling crumb after cutting,
7. dumpling crumb stickiness,
8. overall product impression.

These eight sensory parameters were graded on a 5-point scale, where 5 indicated the best score and 1 the worst (see Supplementary Table S1a for more details). For example, the taste of dumpling could be described as 5 = *very pleasant*, 3 = *less pronounced,* or 1 = *alien* (e.g.*, very bitter*). For an easier comparability of the control wheat dumpling and 2×7 enriched product variants, partial points of the 8 sensory attributes listed above were summed together under the *Dumpling sensory score*, ranging in limits 8–40 points, and under the *Sensorial acceptability* (0–100%, respectively). On a mentioned 5-point scale, both the calculated parameters the *Dumpling sensory scores* and the *Sensory acceptability* could be interpreted as 5 = *like strongly*, 4 = *like*, 3 = *like nor dislike*, 2 = *dislike*, and 1 = *dislike strongly* (Supplementary Table S1b). The *Sensorial acceptability* parameter is a function of the *Dumpling sensory score*: *Sensorial acceptability* (%) = *Dumpling sensory score* / 40, where 40 means the maximal (ideal) sensory score in points.

Ethical review and approval were not required for this study involving human participants, in accordance with local legislation and institutional requirements. Written informed consent was obtained from all participants.

### 2.5 Determination of *β*-d-glucans

The total content of *β*-d-glucans in the sample was determined using the β-Glucan (Mixed Linkage) set (Megazyme by Neogen®, Ireland). The streamlined method has been successfully evaluated by the AACC (Method 32–23.01), the AOAC International (Method 995.16), and the ICC (Standard Method No. 166). The mature oat grains were suspended and hydrated in three repetitions in a sodium phosphate buffer (pH 6.5), incubated with purified lichenase (enzyme of the EC number 3.2.1.73), and filtered. The aliquot of the filtrate was hydrolysed with *β*-glucosidase (enzyme of the EC number 3.2.1.21). Oxidase/peroxidase reagent was used to examine the glucose obtained, which was subsequently converted to *β*-d-glucans content in the sample and the dry matter.

### 2.6 Determination of proteins

The nitrogen level was determined using the Dumas method. A sample of 200 mg in two replications was weighed into a ceramic boat and placed into the automatic feeder of the TruMac CNS Macro Analyzer (LECO Corp., St. Joseph, MI, United States). After completion, the instrument software calculated the concentration of total N-substances. The total protein content was recalculated using a factor of 5.70 for wheat flour, binary flour mixes, and dumplings and 6.25 for oats according to ICC Standard No. 105/2 (ICC, 1994). The total protein content was converted to dry matter.

### 2.7 Determination of lipids

The lipid content was determined by Soxhlet extraction using n-hexane as the solvent, following ISO 659:2009 (Oilseeds, Determination of oil content, reference method, ISO, 2009) and AACC Method 30–25.01 (Crude Fat in Grain and Grain Products, Soxhlet Extraction, AACC International, 1999), with minor modifications. Each sample was weighed twice, with 5.0 g being used per replicate. The lipids were extracted using a custom-built Soxhlet apparatus and n-hexane as the solvent, with extraction carried out for 5 h and 4 h on two consecutive days. The total lipid content of the sample was determined by calculation in 100% dry matter.

### 2.8 Determination of starch

The starch content was determined in triplicate by the Ewers polarimetric method, following ISO 10520:1997 / EN ISO 10520:2002 (Native starch, Determination of starch content) (ISO, 1997) and ICC Standard No. 123/1 (Polarimetric Method for the Determination of Starch, ICC, 1994). The method is based on partial hydrolysis of starch using hydrochloric acid, after which the optical rotation of the obtained solution is measured in a 200 mm cell at 21 °C and recalculated to starch and dry matter content.

### 2.9 Statistical evaluation

All data collected were processed in the Statistica 13.0 software (TIBCO Inc., United States). Data scatter and statistical closeness of the samples was quantified by two-factorial analysis of variance (ANOVA), that is, by Tukey’s HSD test (*p* < 0.05). Within the ANOVA, the strength of the factors ‘*Oat variety*’ and ‘*Oat flour ratio*’, plus their interaction, was also described by a plot of variance components plot. At the same likelihood level, relationships among the contents of nutritional components of the flour and dumplings were described by linear correlation analysis. For the chemical composition of the basic flour threesome and their binary mixes, as well as of the relative steamed dumplings, visualization of the correlation matrix was carried out by Principal Component Analysis (PCA). For dumpling samples only, the links among the type of oat flour, the levels of addition of the oat flour, the chemical composition, and the sensory attributes were explored by clustering – a two-way joining, building up the so-called ‘*heatmap*’. This underestimated clustering method is based on the Z-scores of each input variable, which shows the distribution of values around the variable’s average. Under the condition of normal distribution of the variable, the Z-scores usually exist in the interval <−3; 3>; there are three characteristic cases:

- Z > > +1: strongly above the average, meaning a positive/negative effect of the observed factor,
- Z ≈ 0: near the average, equal to the weak (statistically insignificant) effect of the observed factor,
- Z < < −1: strongly below the average, meaning a negative/positive effect on the considered sample.

## 3 Results

An evaluation of the chemical composition of experimental flour mixtures and steamed wheat dumplings fortified with wholegrain flour from two oat varieties Inovec and Prokop (Ino and Pro), was carried out at seven incremental replacement levels (2.5 to 25.0%) was conducted. As seen in Table 1, the oat varieties were characterized by a statistically significantly higher proportion of dry matter, *β*-d-glucans, lipids, and proteins compared to the wheat sample. On the other hand, wholegrain oat flour had a lower starch content than semi-coarse wheat flour, which formed the basis of innovative food products. Based on the results, the development of an innovative food product incorporating wholegrain oat flour into wheat flour is justified, given the increased content of health-promoting metabolites such as *β*-d-glucans, proteins, and plant-derived lipids. In addition, the lower starch content of oat flour relative to wheat flour results in a reduced starch level in the final product. When combined with the higher proportion of dietary fiber, particularly its soluble fraction, *β*-d-glucans, this compositional profile contributes to a reduced glycemic index. Within the framework of current nutritional guidelines, such a characteristic is considered beneficial and nutritionally desirable.

**Table 1:** Comparison of basic nutritional composition of three cereal raw materials, used for functional reformulation of wheat based yeasted steamed dumplings.

F1 – Flour type	Dry matter (%)	β-D-glucans (%)	Proteins (%)	Lipids (%)	Starch (%)
Oat flour Ino	91.26c	5.60a	18.44b	7.48c	58.86a
Pro	90.72b	6.48a	19.67c	5.93b	59.51a
Wheat flour Commerce	88.77a	0.23b	9.93a	1.83a	76.78b

ANOVA factor *Type of flour*: wholegrain oat flour [with subcategories of the hull-less oat variety Inovec (Ino), and wholegrain flour of the hulled oat variety Prokop (Pro)], and commercial semi-coarse wheat flour. Different letters within each column indicate statistically significant differences (*p* < 0.05, Tukey’s HSD test).

Table 2 provides a summary of the measured values for dry matter, *β*-d-glucans, proteins, lipids, and starch content of flour mixtures and steamed dumplings enriched with varying levels (2.5–25.0%) of two oat varieties. As part of the experiments, an attempt was made to prepare dumplings using both wholegrain oat flour only. According to the assumption, the lack of viscoelastic gluten made it impossible to create a cohesive, non-sticky oat dough.

**Table 2:** Comparison of chemical composition of flour mixtures and steamed wheat dumplings enriched with two oat varieties (three-factorial ANOVA, *p* < 0.05).

Product type	Flour/Dumpling mixture	Oat flour ratio	Dry matter (%)	β-D-glucans (%)	Proteins (%)	Lipids (%)	Starch (%)
Flour	Wheat Flour (WF)	0.0%	88.77cde	0.22a	9.93a	1.83abc	76.78p
Flour mix	WF + oat Ino	2.5%	88.34ab	0.30abcd	10.08ab	2.03abcd	75.93o
		5.0%	88.50abcd	0.35cdef	10.22ab	2.38cde	75.55no
		7.5%	88.20a	0.51ij	10.86de	2.91efg	73.92kl
		10.0%	88.87def	0.49hij	10.67cd	2.67def	75.04mn
		15.0%	88.42abc	0.67l	11.01ef	3.58gh	73.16jk
		20.0%	88.92ef	0.80n	11.50g	4.09h	72.53ij
		25.0%	89.42g	0.95o	12.54h	3.36fgh	72.57ij
	WF + oat Pro	2.5%	88.22a	0.26ab	10.18ab	1.85abc	75.93o
		5.0%	89.23fg	0.41efgh	10.34bc	2.72def	75.76no
		7.5%	88.37abc	0.44fghi	10.72de	1.43a	75.78no
		10.0%	88.47abcd	0.57jk	10.69cde	1.60ab	75.61no
		15.0%	88.71bcde	0.81n	11.36fg	1.88abc	74.66lm
		20.0%	89.20fg	0.93o	11.69g	2.26bcde	74.32lm
		25.0%	89.01ef	1.23p	12.37h	2.41cde	73.00ij
Dumpling	Wheat dumpling (WD)	0.0%	91.87i	0.27ab	13.91i	5.46ij	72.23hi
Dumpling mix	WD + oat Ino	2.5%	92.37jk	0.30abcd	13.86i	5.97jkl	72.24i
		5.0%	92.11ij	0.36cdef	14.47jkl	5.03i	72.25i
		7.5%	91.46h	0.37def	14.37jk	6.32lm	70.40e
		10.0%	92.91mn	0.43efghi	14.73lmn	6.96m	70.79efg
		15.0%	92.36jk	0.57jk	14.92mn	6.36lm	70.52ef
		20.0%	93.27n	0.76mn	14.51jkl	10.69o	67.31cd
		25.0%	92.99mn	0.80n	15.05n	12.13p	65.01a
	WD + oat Pro	2.5%	92.50jkl	0.27abc	14.19ij	5.63ijkl	72.41ij
		5.0%	91.82hi	0.35bcde	13.93i	6.25klm	71.29fg
		7.5%	91.83hi	0.40efg	14.46jkl	5.52ijk	71.45gh
		10.0%	92.93mn	0.46ghi	14.67klm	6.94m	70.86efg
		15.0%	92.64klm	0.57jk	14.79lmn	9.35n	67.93d
		20.0%	92.84lm	0.62kl	14.77lmn	10.70o	66.76bc
		25.0%	92.35jk	0.69lm	14.95mn	10.44o	66.28b

ANOVA factors: *Product type* (flour or steamed dumpling); *Flour/Dumpling mixture* – WF (100% wheat flour, control), WF + oat Ino (hull-less oat variety Inovec), WF + oat Pro (hulled oat variety Prokop); WD – Wheat dumpling (100% wheat flour, control); WD + oat Ino / Pro – Dumpling samples enriched with corresponding oat flour; *Oat flour ratio* – a percentage substitution of WF by weight. Data are expressed as mean values (% w/w, dry basis). Different letters within each column indicate statistically significant differences (*p* < 0.05, Tukey’s HSD test).

Statistically significant differences are denoted by letter-based groupings (*p* < 0.05). The dry matter content of the flour mixes ranged from 88.20 to 89.42%, and in the case of the dumplings, the range was from 91.46 to 93.27%. A minor increase in dry matter was observed with increasing oat levels in both oat varieties, particularly in cooked products, likely to reflect higher levels of fiber and thermal dehydration. The highest values were recorded for dumplings with 20% oat flour (93.27% in D20Ino), indicating reduced water retention or greater water loss during cooking.

As anticipated, the level of *β*-d-glucans increased in proportion to the degree of oat enrichment. The baseline wheat control contained only 0.22–0.27% of the metabolite, while the highest levels reached 1.23 and 0.95% in flour mixtures with 25% Prokop and Inovec, respectively. After the steaming process, the dumpling formulations exhibited notable levels of *β*-d-glucans (up to 0.80% in D25Ino and 0.69% in D25Pro), suggesting moderate thermal stability of this soluble component of dietary fiber during hydrothermal treatment (a steam cooking). Overall, Inovec samples consistently exhibited higher levels of *β*-d-glucans than Prokop at equivalent substitution levels, thereby corroborating earlier findings regarding genotypic variations in the composition of the outer layer.

The addition of oats in binary flour mixes results in elevated protein concentrations in both the flour and dumpling samples. The highest values were observed in steamed wheat dumplings fortified with 25% oat flour: 15.05% in D25Ino and 14.95% in D25Pro, compared to 13.91% in the wheat-only control WD. This phenomenon is attributable to the inherent higher protein content of oat whole grain, a property that is particularly evident in Inovec-enriched formulations. The observed increases are consistent with the findings of previous studies that have highlighted oats as a promising protein fortifier in cereal-based matrices.

The addition of oats resulted in a significant increase in lipid content, particularly in steamed dumplings. The lipid content of the dumplings increased from 5.46% in the control group to as high as 12.13% in D25Ino and 10.44% in D25Pro. The increase was more pronounced in samples enriched with Inovec, consistent with the higher oil content of hull-less oats. These results confirm oats as a significant contributor to lipid enrichment in composite cereal systems.

A consistent inverse relationship was observed between the inclusion of oats and the starch content of the analyzed samples. The substitution of wheat flour with oat flour resulted in a reduction in the starch fraction in both the dough and the cooked food products. The starch content of the control dumpling was found to be 72.23%, which declined to 65.01% in D25Ino and 66.28% in D25Pro. This reduction can be attributed to the lower starch density of oats compared to wheat, as well as to the displacement effect caused by other macronutrients, such as proteins, lipids, and fiber. It is noteworthy that elevated substitution levels of Inovec resulted in a more pronounced decline in starch content, thereby corroborating the more substantial compositional shift observed in hull-less varieties.

This dataset confirms that fortification with oat flours significantly modulates the macronutrient composition of both raw dough and steamed wheat dumplings. It is noteworthy that increases in *β*-d-glucans, proteins, and lipid contents were accompanied by a dilution in starch concentration, reflecting the distinct biochemical profile of oats. The Prokop cultivar demonstrated efficacy in the process of *β*-d-glucans enrichment, while Inovec exhibited superiority in terms of protein and lipid levels. The findings indicate that the selection of oat varieties and substitution levels are pivotal parameters in the customization of the nutritional profile of cereal-based steamed foods. This emphasizes the necessity of these parameters in the formulation of fiber-rich, functional food products.

A correlation analysis was conducted to assess both the effects of formulation and the retention of the main nutritional components throughout the production chain (Table 3). The proportion of oat flour in the recipe (*Oat flour ratio*) exhibited strong and significant correlations with the final composition of the steamed wheat dumplings, most notably for *β*-d-glucans (*r* = 0.972), lipids (*r* = 0.919), and proteins (*r* = 0.831), while the starch content showed a strong inverse relationship (*r* = −0.954). These findings confirm the direct impact of oat fortification on the nutritional profile of the final product and demonstrate the effectiveness of formulation as a tool for dietary optimization.

**Table 3:** Correlation analysis of oat enrichment of wheat flour (flour mixes) and nutritional composition of steamed dumplings.

Variable		Oat flour ratio^1^	Dumpling composition				
			*– Dry matter*	*– Starch*	*– β-D- glucans*	*– Proteins*	*– Lipids*
Oat flour ratio^1^		1	0.572***	−0.954***	0.972***	0.831***	0.919***
Flour composition	*Dry matter*	0.595***	0.413*	−0.676***	0.597***	0.317*^ns^*	0.704***
	*Starch*	−0.881***	−0.416*	0.814***	−0.886***	−0.715***	−0.764***
	*β-d-glucans*	0.968***	0.463*	−0.934***	0.905***	0.786***	0.887***
	*Proteins*	0.973***	0.469***	−0.964***	0.930***	0.794***	0.917***
	*Lipids*	0.500**	0.291*^ns^*	−0.443*	0.580***	0.316*^ns^*	0.434*

*N* = 30	*^ns^ p* > 95%	* *p* = 95%	* *p* = 99%	*** *p* = 99.9%
*r*_***crit***_	< 0.361	0.361	0.463**	0.570

1 Oat flour ratio – wheat flour replacement from 0.0, 2.5, 5.0, 7.5, 10.0, 15.0, 20.0 to 25.0%; ^ns^ – not significant. *Significant at *p* = 95%, **p = 99%.

Concurrently, the correlations between the nutrient concentrations in flour mixtures and the steamed dumplings offer insight into the processing stability of the individual macronutrients. A strong correlation was observed for *β*-d-glucans (*r* = 0.905), proteins (*r* = 0.794), and starch (*r* = 0.814), indicating that the majority of compositional characteristics were preserved during the processes of mixing, fermentation, and steaming processes. The elevated level of correlation between *β*-d-glucans and the thermal resilience of this functional fiber under mild hydrothermal processing conditions is of particular interest.

Increasing the proportion of oats in the product has a clear beneficial effect on its nutritional value. However, the final dietary outcome is influenced not only by the formulation of the product but also by the extent to which specific nutrients can withstand the processing involved. This dual approach thus offers a more complete framework for functional food development, linking ingredient engineering with process reliability. The correlation analysis confirms that increasing the proportion of oat flour is a reliable strategy for enhancing the content of *β*-d-glucans, proteins, and lipids in traditional steamed wheat dumplings while predictably reducing starch content. The strong statistical relationships further support the feasibility of targeted functional food design based on controlled ingredient manipulation.

**Figure 1:**
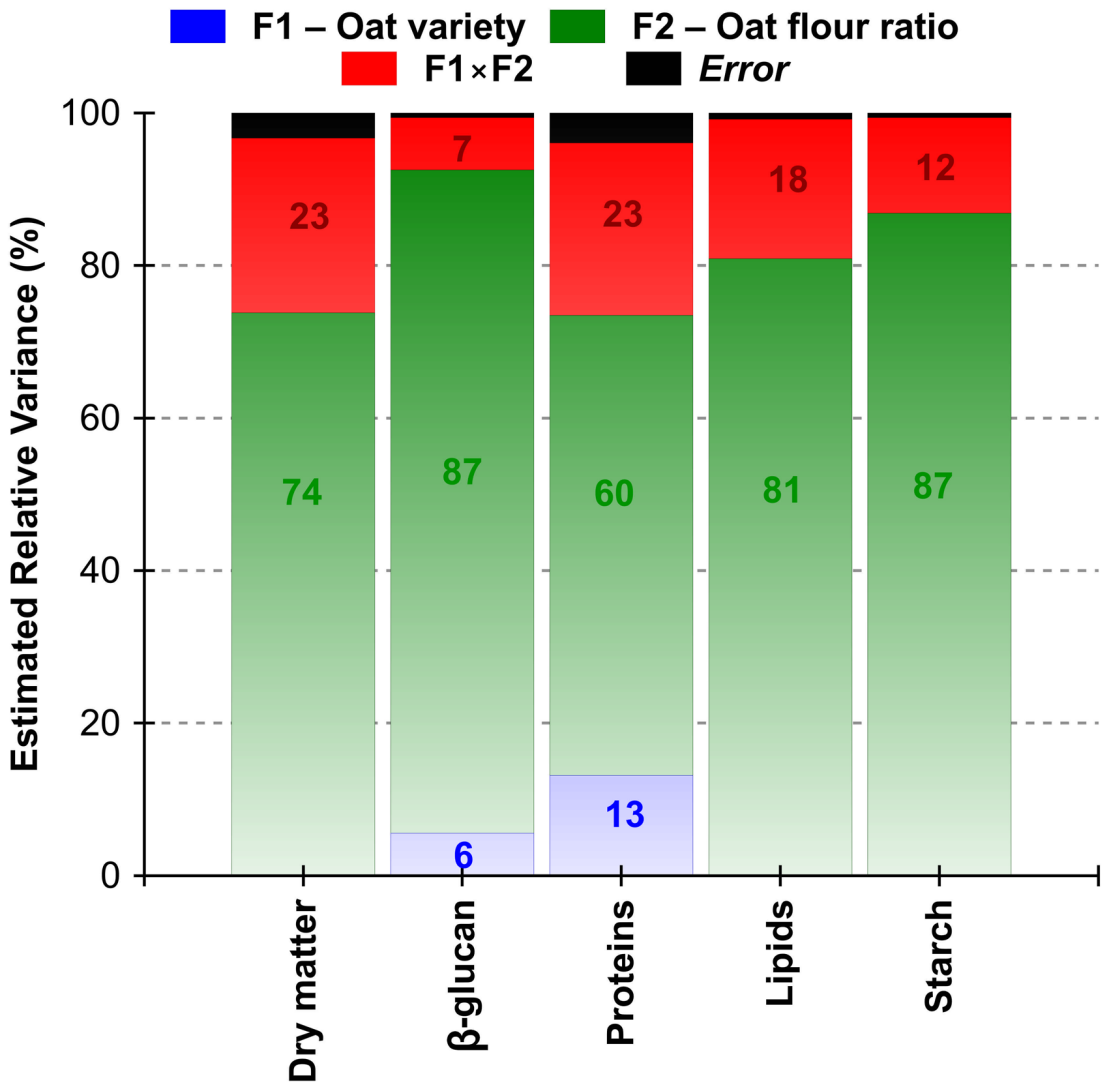
Estimated contribution of factors *Oat variety*, *Oat flour ratio*, and their interaction to the total variance (%) in selected nutritional parameters of steamed wheat dumplings. Factors F1 – *Oat variety* (Inovec vs. Prokop); F2 – *Oat flour ratio* (2.5–25.0% substitution level); F1 × F2 – interaction effect; Error – unexplained residual variance (two-way ANOVA, *p* < 0.05).

The estimated relative variance percentage derived from the two-factorial analysis of variance (ANOVA; Figure 1) demonstrated that the *Oat flour ratio* was the predominant factor influencing the composition of the steamed dumplings in all parameters. For instance, 87% of the total variance in *β*-d-glucans content was explained solely by the level of oat inclusion factor, with minimal contributions from the second factor *Oat variety* (6%) and their interaction effect (7%). Analogous trends were observed for starch and lipid contents, where the flour ratio represented 87 and 81% of the variance, respectively. Conversely, the protein content exhibited a more balanced influence, with 13% of the variance attributed to cultivar differences and 60% to the wheat-oat flour ratio. These findings suggest that while some nutrients (*β*-d-glucans, starch) are primarily formulation-driven, others (proteins) are modulated by both genotype and dose, highlighting the importance of variety/genotype selection in protein-rich applications.

Principal component analysis (PCA) was performed to visualize the relationships between formulation variables and nutritional components of both flour mixes and the dumplings. Owing to the linearity in the input data (in composition of both product types), the initial two principal components accounted for 95.3% of the total variance (PC1 73.8%, PC2 21.5%); further, the remaining two PC covered the rest (4.7–4.0% by PC3, 0.7% by PC4).

**Figure 2:**
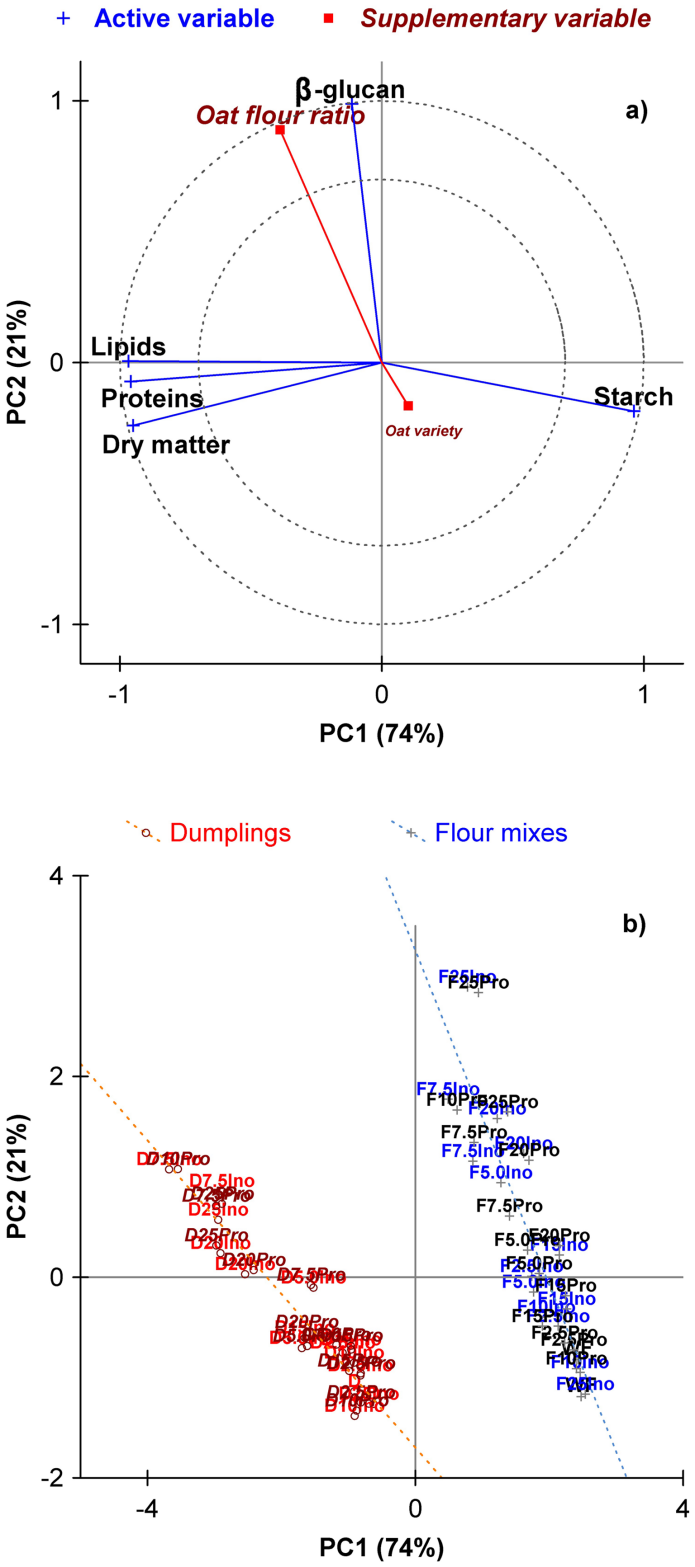
Principal component analysis of the factors *Oat variety* and *Oat flour ratio* effect on the nutritional composition of binary flour mixes and steamed wheat dumplings. **(a)** plot of variable loadings, **(b)** plot of case scores. Supplementary – passive variables (not included in the calculated PCA model): *Oat flour ratio* (percentage of oat substitution) and *Oat variety* (Inovec vs. Prokop).

The PCA plot of variables (Figure 2a) shows that the contents of *β*-d-glucans, proteins, lipids, and dry matter are positively associated, closely aligned with the vector representing the oat flour ratio in both flour mixes and binary dumplings (Figure 2b). This suggests that increasing inclusion of oat flour enhanced all of these components simultaneously. On the contrary, the starch content is situated in the opposite quadrant, reflecting its dilution as oat flour replaced wheat flour. The vector representing the supplementary (passive, explaining) variable *Oat variety*, exhibited a comparatively smaller effect, located close to the plot center.

This finding suggested that, while there are some differences between the Inovec and Prokop oat varieties, they are negligible relative to the impact of the substitution level. These observations are consistent with the findings of the variance analysis, which attributed most of the variance to the oat flour ratio among all variables.

In the PC1 × PC2 plane, the separation of the nutritional compounds into 3 groups predestined the positions of the binary flour mixes and the relative dumplings. Flour mixes and wheat-oat dumplings were divided up along the PC1 axis according to chemical composition – In average, higher contents of dry matter, proteins, and fat were determined, and vice versa of *β*-d-glucans and starch in the latter group. The second PC could be interpreted as the ratio of oat flour present in the flour mixture or steamed dumpling; however, there was no visible unequivocal tendency in the positioning of the samples.

**Figure 3:**
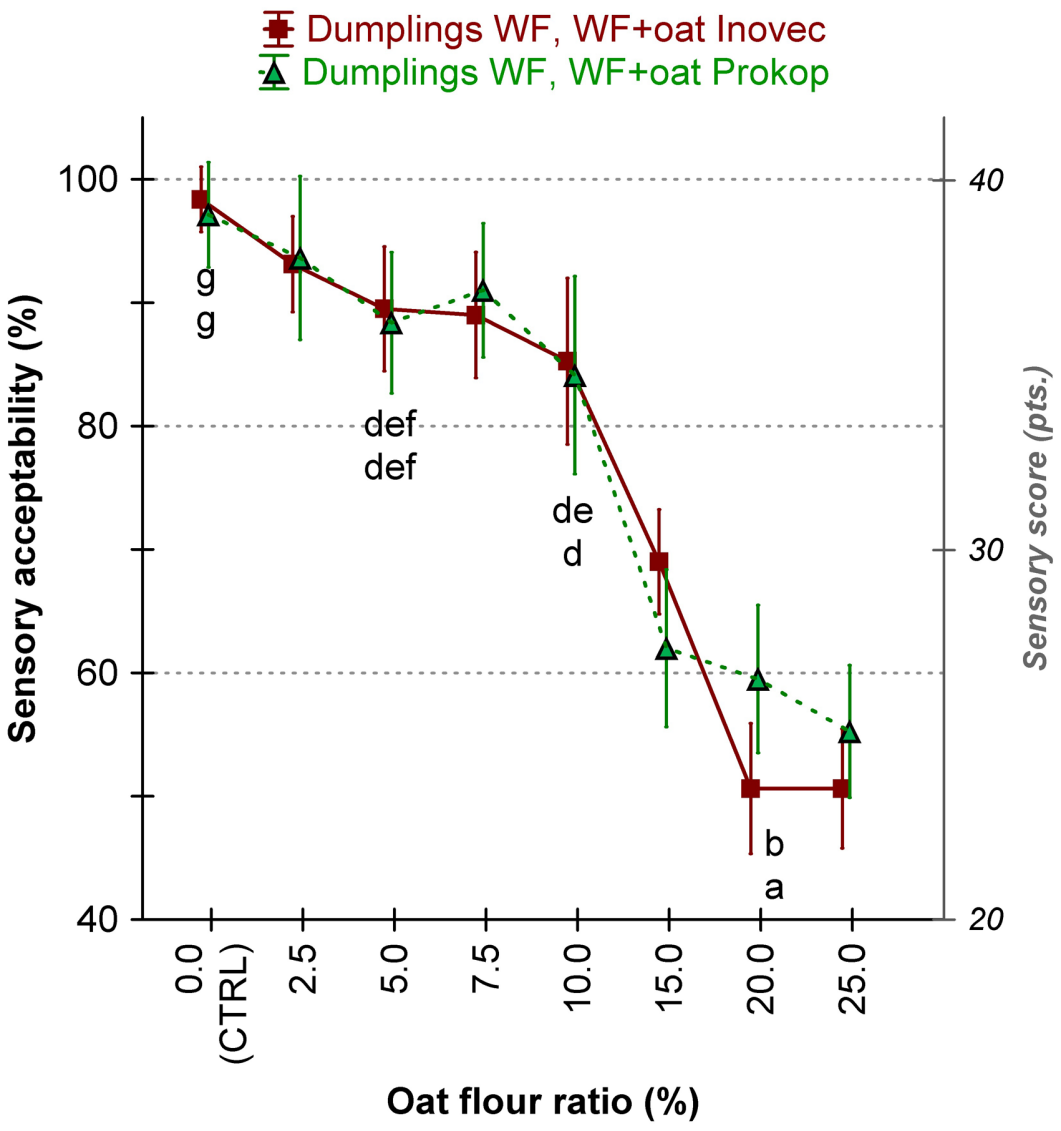
Effect of two oat varieties Inovec and Prokop, and the oat flour portion on the sensorial acceptability of the steamed wheat dumplings (WF –wheat flour; Dumpling WF, 0.0 CTRL– non-enriched control wheat dumpling).

Concerning the highest possible sensory score (the best, the optimal) of dumplings equal to 40 points, the overall sensorial scores 17–40 pts. were recalculated as a percentage of the sensorial acceptability (i.e., in a range 43–100%; Figure 3). A statistically significant diminishing in the pleasant feeling of the 10 panelists is observed during a stepwise consumption of wheat-oat dumpling sets, regardless of the oat variety tested. As presumed, the higher the fortification level, the worse the sensorial acceptability was registered. The main reasons for such negative scoring were especially the color of the crust and crumb, and the smell of the warm food product.

**Figure 4:**
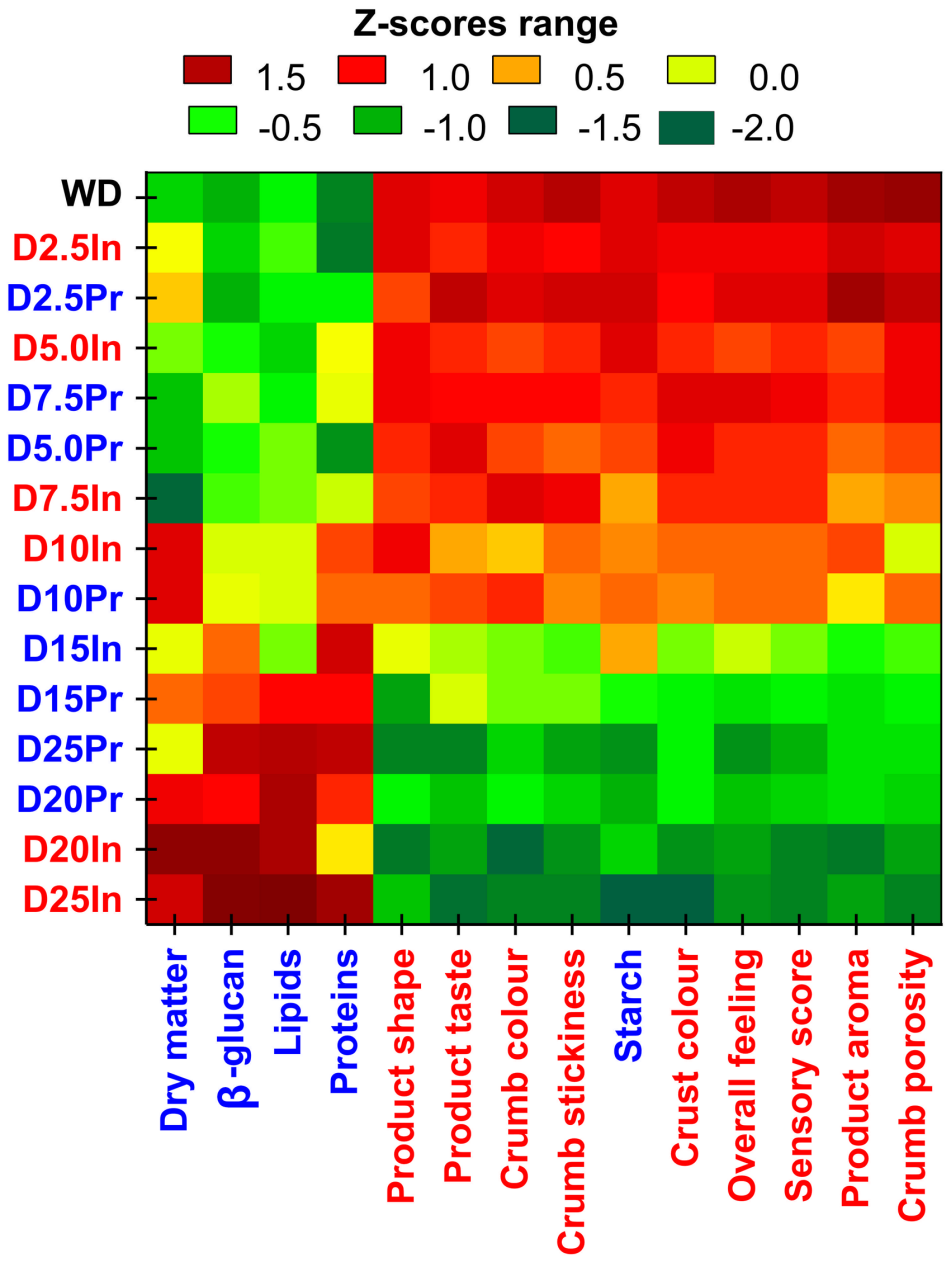
Two-way joining heatmap of nutritional and sensory characteristics of steamed wheat dumplings enriched with oat flour (2.5–25.0%) from two oat varieties (Inovec and Prokop). The heatmap displays Z-score – standardized values for chemical composition traits (blue labels) and sensory parameters (red labels) across steamed wheat and wheat-oat dumpling samples. Rows represent individual formulations; columns denote measured characteristics. Color coding indicates relative performance: Red (Z > +1) strongly above average; Yellow (Z ≈ 0) near average; Green (Z < −1) strongly below average. Dumpling samples coding: WD – wheat control (0% oat); D2.5–D25 – dumpling samples with 2.5–25.0% oat flour; Oat varieties: Pr – Prokop; In – Inovec. Two-way joining highlights clusters of formulations and characteristics with similar behavior.

The sensory properties of the steamed wheat dumplings enriched with varying levels (2.5–25.0%) of two oat varieties were evaluated by two-way joining analysis (Figure 4). This approach integrates nutritional and sensory dimensions into a clustered heatmap, enabling the identification of formulation-related sensory shifts across multiple descriptors.

Samples exhibiting lower levels of oat substitution (2.5–10.0%), particularly those containing D2.5Pr, D5.0Pr, and D7.5Pr, demonstrated consistently high performance across a range of main sensory characteristics, including product shape, taste, crumb color, and overall sensory score. The samples exhibited a close clustering with the wheat control (WD), suggesting minimal sensory deviation from the reference product. It should be noted that these variants also maintained optimal nutritional enrichment, as evidenced by elevated levels of *β*-D-glucans and proteins, indicating a favorable range in the formulation window.

In contrast, samples that showed elevated inclusion levels (≥15.0%), specifically D20In and D25In, exhibited substantial sensory degradation, as indicated by the ANOVA whisker graph of the sensorial acceptability (Figure 1). The samples in question were found to demonstrate substandard performance in some important properties, including product aroma, crumb porosity, overall feel and taste. This is evidenced by the predominance of green field in the Z-score plot, indicating Z-scores below −1. These alterations are presumably associated with matrix destabilization, a consequence of the excessive dilution of the gluten network, which is further exacerbated by the presence of bran fractions of oat flour, leading to the development of a more pronounced bitterness and a denser crumb texture.

As demonstrated in Figure 4, a marked decline in product taste and aroma was observed beginning with 15.0% oat addition, particularly in Inovec-based formulations. Crumb porosity and stickiness were adversely affected in high-substituted samples (D20In, D25In, D25Pr), correlated with starch dilution and fiber-induced water retention, which can interfere with gas cell expansion and steam stability. Observations were made regarding changes in the color of the crust and crumb. It was determined that moderate additions of oats resulted in a darker and more esthetically pleasing appearance, while excessive levels led to undesirable gray-brown tones.

Although both varieties of oat influenced sensory outcomes, Inovec-based samples exhibited a more pronounced decline in acceptability at higher substitution levels compared to their Prokop-containing counterparts. This suggests that the hull-less oat variety Inovec, despite its higher nutritional potential (e.g., in lipids and proteins), may introduce stronger off-flavors or more pronounced structural changes when used in excess.

Sensory analysis indicates that the incorporation of oat flour in steamed dumplings results in an improvement of nutritional quality by up to approximately 10.0 to 15.0% substitution, without compromising overall sensory acceptance. Beyond this threshold, a decline in the quality of the steamed dumplings becomes increasingly evident, with negative changes in taste, texture, and visual appeal. The findings of this study indicate that moderate oat enrichment may be a viable and acceptable solution to enhance the nutritional quality of conventional cereal-based foods.

## 4 Discussion

The presented work is a model example of how a traditional and nutritionally less valuable side dish, white wheat flour-based steamed dumplings, can be transformed into a functional food. Our findings, supported by existing scientific literature [e.g., (10, 18, 19)], indicate that oats are a suitable primary raw material for the development of functional foods, due to their content of metabolites with well-documented functional and biological benefits for human health, such as *β*-d-glucans (8, 10, 11, 13–15, 18), proteins (21, 23), and lipids with an appropriate portion of oat-derived polar lipids (21). Beneficial for the development of functional foods are also secondary metabolites observed in oat wholegrain flour (19) with proven antioxidant activity, such as avenanthramides typical in oats (20).

Our results unequivocally demonstrate that fortification of wheat flour with oat significantly modifies the macronutrient composition of both raw flour mixture mixes and finished steamed dumplings. Compositional shifts are primarily dictated by the proportion of oat flour in the mixture, a fact confirmed by both correlation and variance analyses.

Strong positive correlations between the oat flour ratio and the main nutritional components, particularly *β*-d-glucans (*r* = 0.972), lipids (*r* = 0.919), and proteins (*r* = 0.831), confirm the compositional leverage of oat fortification. Concurrently, the significant negative correlation with starch content (*r* = −0.954) underscores the displacement effect associated with the increase in the inclusion of oats. The observed patterns were consistent across both hull-less and hulled variety of oat (Inovec and Prokop, respectively) and remained largely stable through thermal processing. This stability was evidenced by significant flour-to-dumpling correlation coefficients for *β*-d-glucans (*r* = 0.905), proteins (*r* = 0.794), and starch (*r* = 0.814).

The traditional side dish was enriched with nutrients, including *β*-d-glucans, proteins and lipids, which may indicate a useful synergistic effect in the development of multifunctional foods. The co-presence of *β*-d-glucans with proteins and lipids enhances the hypocholesterolemia impact of oats (25). A synergistic effect is also observed in the functional role of oat *β*-d-glucans as an emulsifier, thickener, stabilizer, and gelling agent. These physical interactions, especially when coupled with oat lipids and proteins, enable the development of stable, functional food products (26).

The reduction in starch content resulting from the incorporation of oat flour into wheat-based dumplings can be regarded as a positive step toward the development of foods with reduced starch levels and consequently lower starch intake by consumers. In combination with the higher dietary fiber content and its functional components, this reduction contributes to a lower glycemic index of the product. Current research suggests that cereal-based foods formulated with reduced digestible starch content and increased non-digestible fiber, such as resistant starch or *β*-d-glucans, can lower caloric density and improve glycemic control, offering significant advantages in the development of functional foods intended for weight management and metabolic health (22, 27, 28). Controlled interventions and cohort studies consistently show that cereal-based diets – especially those high in whole grains and low in rapidly digestible starch – improve insulin sensitivity, reduce postprandial insulin and triglyceride responses, and lower the prevalence of metabolic syndrome (18, 23, 29, 30).

The analysis of variance further corroborated the formulation-centric nature of the observed changes. For all nutrients that were the subject of evaluation, the Oat flour ratio factor (F2) accounted for the majority of the variance, ranging from 60% for proteins to 87% for *β*-d-glucans and starch, respectively. The interaction with the F1 – *Oat variety* (F1 × F2) explained a moderate proportion of the variation (12–23%), while the oat variety alone contributed minimally to most traits, except for proteins (13%). This finding supports the conclusion that the level of oat inclusion is the primary driver of nutritional modification, with variety / genotype playing a secondary role in certain parameters. Bread with varying levels of oat flour / bran levels showed that the oat inclusion primarily influenced volume, texture, and *β*-d-glucans content, more than the source of oat (31). Research on 20 oat cultivars found although the genotype affected flour traits, bread quality depended more on the oat levels and processing conditions (31). A study with 5–25% oat flour in wheat bread showed that higher oat levels boosted fiber, *β*-d-glucans, and lipids, with minimal impact of the oat genotype (32).

The PCA plot of variables (Figure 2a) confirmed and visually summarized these relationships: the vector representing the oat flour ratio was strongly aligned with increases in *β*-d-glucans, lipids, and protein content, while starch projected in the opposite direction. It is notable that the factor of oat variety exerted only a negligible influence, demonstrating a tendency to gather near the origin. This observation serves to reinforce the dominant impact of dosage over genotypes.

Furthermore, the hierarchical clustering by the heatmap demonstrated a distinct grouping of compositional variables. Close clustering of *β*-d-glucans and proteins indicates that their enrichment proceeds in parallel, a phenomenon that is likely attributable to shared compositional origins in the outer layers of the oat grain (6, 17). The presence of dry matter in this cluster indicates its dependence on fiber and lipid fractions. Conversely, starch exhibited an isolated, distant cluster, indicative of its inverse response to oat supplementation.

These results agree with those reported in previous studies reporting on the role of *β*-d-glucans and protein fractions in the functional enhancement of cereal-based matrices (32–34). The increase in lipid content observed in formulations derived from the Inovec variety is consistent with previously reported varietal differences in oil-rich groats (35). In addition, the thermal stability of *β*-d-glucans observed in this study is consistent with earlier findings on their resilience to hydrothermal processing (8, 15, 36), thereby supporting their bioactivity retention in ready-to-eat foods (36).

From a technological standpoint, the findings substantiate that fortification with oats constitutes a viable strategy for augmenting the content of functional ingredients in steamed dumplings. However, formulation thresholds must be balanced against sensory and textural acceptability. Because when developing an innovative food, it is important not only the nutritional value and biological activity, but also the sensory acceptance by consumers (37, 38).

Texture degradation and the emergence of undesirable flavors were noticeable when oat inclusion levels were higher than 15.0%. Studies show that using more than ~15.0% oat flour often results in increased hardness, chewiness, stickiness, and graininess, due to the gluten structure being diluted and bran components being present. For instance, breads containing 10.0–50.0% oat flour had noticeably firmer and drier crumbs, as well as lower sensory scores, when the oat flour content was ≥ 30.0% (39). These effects are likely to be the result of a dilution of the gluten structure and the bitter components in the bran layer. The addition of oats increases water absorption and dough stability in rheological tests up to a 25.0% inclusion rate, reflecting the hydration properties of oat fibers. However, overuse may also make doughs denser and less extensible (40, 41). Consumer panels rated wheat-oat breads with 10.0–20.0% oat content the highest, whereas replacements above ~30.0% caused a sharp decline in overall acceptability due to textural and taste defects caused by oat particles and bitter bran compounds (42, 43). This observation may be indicative of the higher levels of polyphenols and saponins present in the hull-less oats (35), which are known to contribute to the presence of bitter or grassy notes in the final food product. Wheat loaves enriched with isolated *β*-glucan (Barliv^™^ barley beta-fiber) were not acceptable not only from a sensory point of view, but also due to unsuitable technological parameters (44).

The comprehensive analysis of the dataset confirms the effectiveness and statistical reliability of improving the functional properties of wheat-based products through oat flour incorporation. The optimal balance between nutritional benefit and sensory acceptability appears to lie between 7.5 and 10.0% substitution. The selection of oat genotypes can offer additional refinement, with Inovec demonstrating benefits in terms of lipid and protein enrichment, and Prokop in *β*-d-glucans enhancement.

## 5 Conclusion

This study takes a novel approach to functional cereal-based foods by examining the nutritional impact of oat fortification in yeasted steamed wheat dumplings. This traditional Central European dish has largely been overlooked in large part in terms of dietary fiber enhancement. Unlike previous studies, which focused on dry or baked cereal matrices, this study demonstrates that oat *β*-d-glucans and related nutrients remain stable during fermentation and steaming. The observed enrichment in *β*-d-glucans, proteins, and lipid content, in conjunction with a predictable decrease in starch, confirms the feasibility of tailoring the nutritional composition of moist cereal-based foods through controlled formulation strategies.

This study evaluates two oat varieties across a gradient of substitution levels to provide new insights into how ingredient selection and formulation influence the nutritional profile of steamed cereal-based steamed foods. Consistent enrichment and retention of *β*-d-glucans, proteins, and lipids after fermentation and steaming highlights the practical potential of oat-enriched wheat dumplings as a familiar source of beneficial substances such as proteins and dietary fiber components. These findings broaden the scope of *β*-d-glucans-fortified cereals beyond traditional baked goods, offering a promising approach to developing functional foods based on traditional culinary practices. Based on the nutritional and sensory outcomes observed, an inclusion level of oat flour between 7.5 and 10.0% appears optimal for achieving functional benefits without compromising the acceptability of the product.

Nevertheless, the limitations of this study should be considered. While the *β*-d-glucans content of yeasted steamed dumplings was assessed quantitatively, information regarding the molecular weight and structural integrity was not obtained. These factors may influence their physiological effects and compliance with health claims according to the EU. Further research on the structure and function of *β*-d-glucans after processing could help to clarify their molecular characteristics and digestibility. Studies focusing on the effects of *β*-d-glucans on gut microbiota could also help to clarify their health benefits. Testing oat-enriched formulations in other cereal-based foods would help to evaluate their broader potential. This study provides a foundation for the development of new types of fiber-rich, heat-processed foods containing oat ingredients. This work demonstrates that traditional cereal-based foods can be effectively transformed into functional products through a targeted formulation, offering a promising avenue for improving public health through familiar dietary patterns.

## Data Availability

The original contributions presented in the study are included in the article/Supplementary material, further inquiries can be directed to the corresponding authors.
